# Parental stress and physical violence against children during the second year of the COVID-19 pandemic: results of a population-based survey in Germany

**DOI:** 10.1186/s13034-023-00571-5

**Published:** 2023-02-20

**Authors:** Alina Geprägs, David Bürgin, Jörg M. Fegert, Elmar Brähler, Vera Clemens

**Affiliations:** 1 Hospital of Child and Adolescent Psychiatry/Psychotherapy, University of Ulm, Ulm, Germany; 2 Child and Adolescent Psychiatric Research Department (UPKKJ), Psychiatric University Hospitals, University of Basel, Basel, Switzerland; 3 Department for Psychosomatic Medicine and Psychotherapy, University Medical Center of Johannes Gutenberg University of Mainz, Mainz, Germany; 4 Integrated Research and Treatment Center Adiposity Diseases, Behavioral Medicine Unit, Department of Psychosomatic Medicine and Psychotherapy, Leipzig University Medical Center, Leipzig, Germany

**Keywords:** Parental stress, Physical violence, Child maltreatment, COVID-19 pandemic, Adverse childhood experiences

## Abstract

**Background:**

Parents and caregivers belonged to those with the highest burdens during the COVID-pandemic. Considering the close link between parental stress and child maltreatment, identifying families with high parental stress is of utmost importance to prevent violence against children. Within this study, we thus aimed to investigate the interplay of parental stress, changes in parental stress, and physical violence against children during the second year of the COVID-pandemic on an exploratory level.

**Methods:**

We conducted a cross-sectional, observational study in Germany from July to October 2021. By using different sampling steps, a representative probability sample of the German population was generated. A subsample of these participants with children under the age of 18 was included for analysis within this study (N = 453, 60.3% females, M_age_ = 40.08; SD = 8.53).

**Results:**

Higher parental stress levels were associated with more physical violence against children, higher levels of own experiences of child maltreatment, and mental health symptoms. An increase in parental stress during the pandemic was associated with female sex, the use of physical violence against children, and parental experience of child maltreatment. Parents who have ever used physical violence against their children have been characterized by higher parental stress levels, a stronger increase in parental stress during the pandemic, own experience of child maltreatment, mental health symptoms and sociodemographic characteristics. Higher parental stress levels, a stronger increase of parental stress during the pandemic, having pre-existing psychiatric disorders, and parental experience of child maltreatment predicted an increased use of physical violence against children during the pandemic.

**Conclusions:**

Our results underscore the importance of parental stress for the risk of physical violence against children, more so in times of overall increased stress due to the pandemic and underline the need for low threshold support for families at risk in times of crises.

**Supplementary Information:**

The online version contains supplementary material available at 10.1186/s13034-023-00571-5.

## Background

Since the beginning of 2020, public life in many areas around the world was largely determined by the spread of the novel severe acute respiratory syndrome coronavirus-2 (SARS-CoV-2). Governments around the globe used various restrictions like school closures or contact restrictions to decelerate the growth of COVID-19 cases [1]. These restrictions were effective to mitigate the spread of the virus [2, 3], however also affect mental health and well-being. During the pandemic, a reduced quality of life [4–6], an increase in mental health problems [7–10], as well as higher levels of stress and distress [7, 11, 12] were shown. Taken together, research has broadly shown the various negative consequences of the COVID-pandemic, especially concerning mental health.

Restrictions to prevent the spread of the virus, even if established for the whole population, have varying impact on different subpopulations; as such measures like school and childcare facilities closures have been impacting foremost families. Families and parents have reported various family-specific stressors during the pandemic as an uncertainty about the situation, inability to meet friends and family, loss of daily structures [13], closed childcare institutions, homeschooling, working from home [14], balancing childcare and work duties [15], and not being able to participate in social activities [16]. On the other hand, the pandemic augmented some protective factors like fewer obligations, more time for family [13], and more outdoor activities [15]. However and unsurprisingly in light of the increased amounts of pandemic-related stressors in families [11], studies have shown higher rates of parental stress across countries [17] and increased rates of parental burnout [18]. Summarizing, studies describe changes in daily functioning, specific pandemic-related stressors, and consequently higher levels of stress for families during the pandemic.

Importantly, not all families have been affected equally and different factors have been shown to increase or decrease the risk for pandemic-related burdens for families. As such, higher levels of parental stress during the pandemic have been reported in families with higher number of children in the household [19], younger parental age [20], older child age [21], in single-parent families, and having a child with pre-existing disorders [22]. Compared to fathers, mothers tended to have higher levels of burnout and a stronger decrease in life satisfaction [17], as well as increased levels of stress [16, 18, 19]. Additionally, parental working conditions are important determinants; parental stress has been reported to be higher when the parental work structure has changed since the beginning of the pandemic [22] and increased to parental unemployed and economic pressure [23]. Moreover, lower educational level of parents was shown to be associated with increased stress levels [23]. Besides these structural and work-related factors, parental mental health and psychological factors were demonstrated to have additional influence. As such, higher parental stress during the pandemic has been associated with depressive symptoms in parents [24, 25], symptoms of anxiety [24, 26], less social support [24], and parental stressful life events [27]. Together, studies have shown associations of different structural and working-related factors as well as mental health of parents to increased parental stress during the pandemic.

Parental stress is well-known to impact family dynamics and functioning—more so during times of pandemic. Unsurprisingly studies have thus shown the parent—child relationship to suffer with increasing levels of stress [20], also harsh parenting was shown to increase [20], just as anger towards children [24]. This is important to acknowledge, as increased levels of parental stress during the pandemic coincide with a higher potential of child abuse [25]. Next to this, parental stress was associated to higher behavioral problems of children [28] and an increase in parental stress in families coincides with an increase of adverse childhood experiences (ACEs) [12]. Concerns around child protection were broadly articulated during the pandemic [29], but findings across studies are highly heterogeneous [30, 31]. As such, partly increased [32–35] and partly decreased [36–42] rates of child maltreatment have been reported. Besides these official statistics, studies which directly interview families and parents and that incorporate data from different sources are scarce. The few existing studies show about 2–20% of parents to report increases in using spanking or hitting their children [43, 44]. Studies also show an increased frequency of physical violence against children [44]. Physical violence seems to be associated with parental depression [45, 46], job loss coping [45], employment change, lower income and increased use of discipline with depression and social isolation [46]. Together, due to the heterogenous findings, systematic research about violence during the pandemic is needed [47]. Considering the relevance of parental stress for the risk of physical violence, it is important to investigate parental stress, changes in parental stress and influencing factors on parental stress during the pandemic in order to prevent the use of physical violence against children and it`s severe long-term consequences. While in Germany, the lockdown took place mainly during a very early phase of the pandemic (March–May 2020) and during winter 20/21 (November 2020–April 2021), the consequences of these measures have not ended with relaxation of measures. In contrast, mental health problems in children have even continued to increase between the first lockdown in spring 2020 and the second lockdown in winter 20/21, and remained on a stable high level until autumn 2021 [48]. School closures took mainly place in the above mentioned phases of lockdown during spring 2020 and in the beginning of 2021. Although varying between the different federal states of Germany, all in all, Germany was the country with the second most frequent school closures in Europe [49].

Within this study, we aimed to investigate parental stress and changes in parental stress since the beginning of the pandemic in a subsample of a German population-representative sample with children under the age of 18 in summer/autumn 21. Moreover, addressing the close link between parental stress and physical violence, we aimed to assess the interplay between parental stress and the use of physical violence against children during the pandemic and associated influencing factors in order to identify high-risk families. To the best of our knowledge, this is the first study focusing on parental stress and changes in parental stress as well as influencing factors at this later stage of the pandemic. Thus, our analyses have rather an exploratory character.

## Methods

### Sample procedure

The sample was obtained by a demographic consulting company (USUMA, Berlin, Germany). First, ADM (Arbeitskreis Markt- und Sozialforschungsinstitute e.V.) systematic area sampling was used. This covers the entire inhabited area of Germany and is based on the municipal classification of the Federal Republic of Germany. There, around 53,000 areas of Germany are defined electronically, with around 700 private households in each area. Afterwards these 53,000 areas are separated into 1500 regional layers and then into 128 “networks”, containing 258 single sample points, which are proportionate to the distribution of private households in Germany. Second, using a random route procedure, private households were systematically selected. To do so, streets were selected randomly, and every third residence was invited to participate in the study. If there was more than one household in the building, every third household was invited, based on the order of the doorbell name plates, starting left at the bottom. Lastly, if there were several eligible persons for the study in the same household one person was randomly chosen with the Kish-selection Grid technique. Inclusion criteria were being at least 16 years old and speaking sufficient German. The target persons got information about the research background and the procedure and signed informed consent. After signing the informed consent, basic sociodemographic characteristics were asked face-to-face by the research staff in an interview at the residence of the participant. With a researcher nearby to answer possible questions the participants fulfilled a questionnaire. The questionnaires and the demographic data were included into a database without containing identifying information. The survey was conducted between July 28th and October 1st 2021, thus before and at the beginning of the fourth wave of COVID-19 in Germany. During this state of the pandemic there were no school, childcare facilities, or shop closures. While performing the interview, hygiene measures were implemented (wearing mask, keeping distance, disinfecting hands). All in all, 5934 target persons were identified, 5908 were contacted. The main reasons for non-participation were refusal of the selected household to provide information (24.0%), refusal of the target person to participate (13.6%) and failure to contact persons in the household after four attempts (13.4%). While the total sample included N = 2515 participants (utilization rate = 42.6%), for this study, only participants with minor children were included. Thus, our final sample included N = 453 participants. The study was conducted in accordance with the Declaration of Helsinki and was approved by the Ethics Committee of the Medical Department of the University of Leipzig.

### Measures

*Parental stress* was assessed with the German version of the parental stress scale (PSS) [50], originally developed by Berry and Jones [51]. It contains 18 self-rating items (e.g. “Parenting makes me happy”) with a scale from 1 (“Doesn’t apply at all”) to 5 (“completely applies”), having a possible range of 18–90. In our sample, a good internal consistency for the PSS was seen (Cronbachs α = 0.91). In the analysis the sum score was used, with higher values meaning higher parental stress. *Change in parental stress during the pandemic* was assessed with an adapted form of the German version of the parental stress scale (original: Schmid et al., 2008); containing 16 self-rating items referring to changes in parental stress compared to the beginning of the pandemic (e.g. “Since the beginning of the pandemic parenting makes me…”) with a scale from − 1 (“happier than before”) to 1 (“less happy than before”). For this modified version of the PSS, an adapted scale was calculated. Negative values represent a negative change in parenting since the beginning of the pandemic while, a value of zero represents no change in parenting during the pandemic and positive values represent a positive change in parenting during the pandemic, with a possible range of – 16 to 16. In our sample a good internal consistency for this pandemic-specific PSS was seen (Cronbachs α = 0.94). *Physical violence* was assessed using a list of different forms of physical violence. Participants who used at least one form were included in the group with use of violence against children. For the analysis, a binary coding with “0” and “1” was used, with “1” representing the group with use of violence. *Own maltreatment experience in childhood* was assessed using the German version of the ICAST [52]. For the analysis a sum score was used, with higher values representing the experience of more different forms of maltreatment, with a possible range from 0 to 4. *Depressive symptoms* were assessed with the Patient Health Questionnaire-2 (PHQ2), a screening tool with a sensitivity of 82% and a specificity of 92% for major depressive disorder [53] providing a good internal consistency (ω = 0.77) [54]. In our sample, a good internal consistency for the PHQ-2 was seen (Cronbach’s α = 0.73). For the analysis the sum score with a possible range from 0 to 6 was used, with higher values representing higher symptom load. *Anxiety* was assessed with the Generalized Anxiety Disorder 2-item (GAD-2), a screening questionnaire with a sensitivity of 86% and a specificity of 83% for generalized anxiety disorder [55], providing a good internal consistency (ω = 0.78) [54]. In our sample a satisfying internal consistency for the GAD-2 was seen (Cronbach’s α = 0.62). For the analysis the sum score with a possible range from 0 to 6 was used, with higher values representing higher symptom load. *Pre-existing psychiatric disorders and somatic disorders* were assessed using a list of disorders. Participants with any (or several) of these disorders were included in the group with pre-existing psychiatric or somatic disorders. For the analysis a binary coding with “0” and “1” was used, with “1” representing the groups with pre-existing disorders. *Employment* was separated into two groups (employed vs. not employed), likewise living in a relationship with mother/father of youngest child, having worked from home during the pandemic, having a “system-relevant” job (e.g. working in the supermarket, in the healthcare system, police or another job not having been affected by lockdown), whether the partner has been working from home during the pandemic, partner has had a “system relevant” work and increase of physical violence during the pandemic. For the analysis, binary coding with “0” and “1” was used. *Income* was separated into two groups (income under poverty level vs. Income over poverty level), based on the German poverty level (1176€) [56]. For the analysis, a binary coding with “0” and “1” was used, with “1” representing the group above the poverty level.

### Statistical analysis

The aim of this study was, first, to investigate parental stress and factors associated to parental stress during later stages of the pandemic, second, to investigate factors associated to changes in parental stress, third, factors associated to the use of physical violence against children, and last, factors associated to change in the use of physical violence against children during the pandemic. All analyses were performed using SPSS version 28. In the regression analyses parental stress and change in parental stress during the pandemic were used as dependent variables. Depressive symptoms, anxiety, age of first child, pre-existing somatic disorder, pre-existing psychiatric disorder, employment, sex, use of physical violence against children, income under poverty level, living in relationship with father/mother of youngest child, own maltreatment experience in childhood, system-relevant job, working from home, system-relevant job of the partner and if the partner works from home were included as independent variables. For each regression model, homoscedasticity, multicollinearity, normal distribution of residuals and independence of residuals were checked (see Additional file 1). Due to heteroscedasticity, robust standard errors and p-values were calculated with a heteroscedasticity-Consistent 3 (HC3) procedure in sensitivity analyses. As no meaningful differences to the original p-values were observed, results of the regression models with non-standardized standard errors were presented. We used listwise deletion to handle missing data. Group comparisons were performed with t-Tests or Chi^2^-Tests, depending on the included variables. As our approach was exploratory, we didn`t correct for multiple comparisons. P-levels are considered as statistically significant at 0.05.

## Results

The final sample included 453 participants, including 273 (60.3%) women. The mean age of participants was 40.08 years (SD = 8.53). Detailed sample characteristics are presented in Table 1.

**Table 1:** Sample characteristics (N = 172–453)

Variable	*N*	*%*	
Sex (N = 453)			
Female	273	60.30%	
Employment (N = 441)			
Employed	398	90.2%	
Living in relationship with father/mother of youngest child (N = 445)			
Yes	352	79.10%	
Working from home (N = 447)			
More than 50% of time	54	12.08%	
Less than 50% of time	53	11.86%	
No working from home	340	76.06%	
„System-relevant “ job (N = 452)			
Yes	110	24.34%	
No	302	66.81%	
Not employed	40	8.85%	
Partner working from home (N = 356)			
More than 50% of time	38	10.67%	
Less than 50% of time	49	13.76%	
No working from home	269	75.56%	
Partner in “system-relevant” job (N = 374)			
Yes	78	20.86%	
Income under poverty level (N = 444)			
Yes	63	14.20%	
Pre-existing somatic disorder (N = 450)			
Yes	88	19.60%	
Pre-existing psychiatric disorder (N = 444)			
Yes	63	14.20%	
Use of physical violence against children (N = 453)			
Yes	118	26.00%	
Increase of physical violence during pandemic (N = 109)			
Yes	22	20.20%	
	M	SD	Range
Age (N = 453)	40.08	8.53	19–81
Age of first child (N = 453)	11.41	6.77	0–43
Own maltreatment experience (N = 453)	0.69	1.13	0–4
Depressive symptoms (N = 453)	0.66	1.02	0–5
Symptoms of Anxiety(N = 453)	0.72	0.99	0–5
Parental stress (N = 446)	36.70	11.73	18–75
Change in parental stress during pandemic (N = 431)	− 0.88	3.96	− 14–10

Mean (M), standard deviation (SD) and Range for continuous variables, frequency and percentage for categorial variables

### Factors associated to parental stress

To identify factors associated with parental stress, multivariate linear regression analysis was performed. As predictors, age of first child, pre-existing somatic disorder, pre-existing psychiatric disorder, employment, sex, use of physical violence against children, income under poverty level, living in relationship with father/mother of youngest child, own maltreatment experience during childhood, depressive symptoms and symptoms of anxiety were included. Results are displayed in Table 2. This model explains a significant and substantial proportion of variance in parental stress (R^2^ = 0.36).

**Table 2:** Associations of sociodemographic characteristics, use of physical violence, own maltreatment experiences in childhood, depression, and anxiety with parental stress

Predictor	B	SE	Standardized Beta	P
*Intercept*	36.12***	2.18		<0 .001
Female sex	− 0.48	0.98	− 0.02	0.62
Employment	− 0.10	1.62	− 0.002	0.95
Income under poverty level	− 0.59	1.42	− 0.02	0.68
Living in relationship with father/mother of youngest child	− 3.59 **	1.20	− 0.12**	0.003
Age of first child	− 0.15 *	0.07	− 0.08*	0.04
Pre-existing somatic disorder	− 1.65	1.27	− 0.05	0.19
Pre-existing psychiatric disorder	1.40	1.51	0.04	0.35
Depressive symptoms	3.54***	0.65	0.29***	<0 .001
Anxiety symptoms	1.62*	0.66	0.13*	0.02
Use of physical violence against children	5.70***	1.17	0.21***	<0 .001
Parental experience of child maltreatment	1.61**	0.49	0.15**	0.001

Presented as Beta coefficients (B) and standard error (SE) and standardized Beta coefficients. (R^2^ = 0.36; F(11,414) = 21.81, p < 0.001). (n = 415). *** p < 0.001, ** p < 0.01, * p < 0.05

Younger age of the first child, use of physical violence against children, not living in relationship with father/mother of youngest child, having experienced more own maltreatment in childhood, more depressive symptoms, and more anxiety symptoms were associated with higher parental stress. Having pre-existing disorders, employment, sex, and income were not significantly associated to parental stress (see Table 2).

### Factors associated to change in parental stress during the pandemic

To identify factors associated with changes in parental stress during the pandemic, linear regression analysis was performed. The predictors age of first child, pre-existing somatic disorder, pre-existing psychiatric disorder, employment, sex, use of physical violence against children, income under poverty level, living in relationship with father/mother of youngest child, own maltreatment experience in childhood, depressive symptoms, anxiety symptoms, working from home, system-relevant job, if the partner works from home and system-relevant job partner were included. Results are displayed in Table 3. This model explains a significant and substantial proportion of variance in change in parental stress (R^2^ = 0.17).

**Table 3:** Associations of sociodemographic characteristics, use of physical violence, own maltreatment experiences in childhood, depression, and anxiety with change in parental stress during pandemic

Predictor	B	SE	Standardized Beta	P
*Intercept*	2.25	1.86		0.23
Female sex	0.97*	0.45	0.13*	0.03
Employment	− 1.94	1.35	− 0.08	0.15
Income under poverty level	− 1.44	0.79	− 0.10	0.07
Living in relationship with father/mother of youngest child	− 0.18	1.08	− 0.01	0.87
„System-relevant “ job	0.51	0.56	0.06	0.36
Working from home	0.13	0.54	0.02	0.81
„System-relevant “ job partner	0.42	0.57	0.04	0.47
Partner working from home	0.27	0.53	0.03	0.62
Age of first child	0.06	0.03	0.10	0.09
Pre-existing somatic disorder	0.32	0.61	0.03	0.61
Pre-existing psychiatric disorder	− 0.64	0.77	− 0.05	0.41
Use of physical violence against children	− 2.01***	0.53	− 0.23***	<0 .001
Parental experience of child maltreatment	− 0.69**	0.24	− 0.20**	0.004
Depressive symptoms	0.15	0.33	0.03	0.65
Anxiety symptoms	− 0.26	0.34	− 0.06	0.44

Presented as Beta coefficients (B) and standard error (SE) and standardized Beta coefficients. (R^2^ = .17; F(15,292) = 3.98, p < .001). (n = 293). *** p < .001, ** p < .01, * p < .05

Female sex, use of physical violence against children and having experienced more maltreatment in childhood were associated with worsening in parental stress during the pandemic. No other variables showed significant associations to change in parental stress during pandemic (see Table 3).

### Factors associated to use of physical violence against children

Table 4 displays differences in the core variables and the sociodemographic characteristics between participants who have reported to have ever used physical violence against their children and participants who never used violence in parenting. Parents who reported to have used at least one-time violence are characterized by higher parental stress, a stronger increase in stress during the pandemic, higher risk of having an income under the poverty level, higher age of first child, more own maltreatment experience in childhood, higher risk of having pre-existing psychiatric and somatic disorders and higher depressive and anxiety symptoms. No differences were seen for age, sex, employment and living in a relationship with mother/father of youngest child.

**Table 4:** Differences in core variables between parents who used at least one-time physical violence and parents who never used violence in parenting

Variable	Use of physical violence against children (N = 118, 26.04%)	Never used violence against children (N = 335, 73.96%)		
	N (%)	N (%)	*X*^*2*^(df)	*p*
Sex (N = 453)			3.78 (1)	0.05
Female	80 (29.30%)	193 (70.70%)		
Male	38 (21.11%)	142 (78.89%)		
Employment (N = 441)			0.01(1)	0.94
Employed	104 (26.13%)	294 (73.87%)		
Unemployed	11 (25.58%)	32 (74.42%)		
Living in relationship with father/mother of youngest child (N = 445)			0.38(1)	0.54
Yes	91 (25.85%)	261 (74.15%)		
No	27 (29.03%)	66 (70.97%)		
Income under poverty level (N = 444)			6.19(1)	0.01**
Yes	24 (38.10%)	39 (61.90%)		
No	89 (23.36%)	292 (76.64%)		
Pre-existing somatic disorder (N = 450)			7.19(1)	0.007**
Yes	33 (37.50%)	55 (62.50%)		
No	85 (23.48%)	277 (76.52%)		
Pre-existing psychiatric disorder (N = 444)			9.36(1)	0.002**
Yes	26 (41.27%)	37 (58.73%)		
No	88 (23.10%)	293 (76.90%)		
	M (SD)	M (SD)	*t* (df)	*P*
Age (N = 453)	40.74 (8.73)	39.85 (8.45)	− 0.98 (451)	0.34
Age of first child (N = 453)	12.43 (6.03)	11.05 (6.98)	− 2.06 (235.23)	0.04*
Own maltreatment experience (N = 453)	0.77 (1.19)	0.40 (0.83)	3.5 (240.9)	< 0.001***
Depressive symptoms (N = 453)	1.08 (1.30)	0.52 (0.86)	− 4.33 (154.54)	< 0.001 ***
Symptoms of Anxiety (N = 453)	1.01 (1.11)	0.62 (0.93)	− 3.66 (451)	< 0.001 ***
Parental stress (N = 446)	43.65 (11.57)	34.20 (10.76)	− 8.02 (444)	< 0.001 ***
Change in parental stress during pandemic (N = 431)	− 3.05 (4.71)	− 0.09 (3.32)	6.21 (157.12)	< 0.001 ***

Frequency displayed as n (%), comparisons via X^2^ or t-Test

### Changes in the use of physical violence during the pandemic

In our sample, n = 23 participants (16.30%) reported to have used more physical violence against their children during the pandemic compared to the time before. Table 5 displays the differences in core variables and sociodemographic variables between participants who have used more physical violence during the pandemic and participants who have not used more violence during the pandemic among those participants who have reported to have ever used physical violence against their children. Parents who have used more violence during the pandemic are characterized by higher parental stress, higher increase of parental stress during the pandemic, and more reported own maltreatment experiences in childhood. No differences were seen regarding sex, income, pre-existing psychiatric or somatic disorders, employment, living in a relationship with father/mother of youngest child, depressive symptoms, anxiety symptoms, working from home, system-relevant job, if the partner works from home, system-relevant job of partner, age, and age of the first child.

**Table 5:** Differences in core variables between parents who used more violence during the pandemic and parents who didn´t use more violence during the pandemic

Variable	Increased use of physical violence against children (N = 22, 20.20%)	Not more use of physical violence against children (N = 87, 79.80%)		
	N (%)	N (%)	*X*^*2*^(df)	*p*
Sex (N = 109)			1.91(1)	0.17
Female	18 (23.68%)	58 (76.32%)		
Male	4 (12.12%)	29 (87.88%)		
Income under poverty level (N = 104)			0.15	0.70
Yes	4 (18.18%)	18 (81.81%)		
No	18 (21.95%)	64 (78.05%)		
Pre-existing somatic disorder (N = 109)			0.18(1)	0.67
Yes	6 (23.08%)	20 (76.92%)		
No	16 (9.28%)	67 (80.72%)		
Pre-existing psychiatric disorder (N = 105)			3.99(1)*	0.046
Yes	8 (36.36%)	14 (63.64%)		
No	14 (16.87%)	69 (83.13%)		
Employment (N = 108)			2.62(1)	0.11
Employed	18 (18.37%)	80 (81.63%)		
Unemployed	4 (40.00%)	6 (60.00%)		
Living in relationship with mother/father of youngest child (N = 109)			0.07(1)	0.79
Yes	18 (20.69%)	69 (79.31%)		
No	4 (18.18%)	18 (81.81%)		
“System-relevant” Job (N = 99)			0.57(1)	0.45
Yes	7 (21.21%)	26 (78.79%)		
No	10 (15.15%)	56 (84.85%)		
Working from home (N = 109)			2.11(1)	0.15
Yes	9 (29.03%)	22 (70.97%)		
No	13 (16.67%)	65 (83.33%)		
“System-relevant“ Job partner (N = 89)			0.12(1)	0.73
Yes	6 (25.00%)	18 (75.00%)		
No	14 (21.54%)	51 (78.46%)		
Partner working from home (N = 84)			0.74(1)	0.39
Yes	8 (29.63%)	19 (70.37%)		
No	12 (21.05%)	45 (78.95%)		
	M (SD)	M (SD)	t(df)	*P*
Parental stress (N = 109)	53.45 (11.10)	40.70 (10.11)	− 5.18(107)***	< 0.001
Change in parental stress during pandemic (N = 106)	− 9.09 (2.84)	− 1.61 (3.97)	8.30(104)***	< 0.001
Own Maltreatment experience (N = 109)	2.45 (1.37)	0.99 (1.23)	− 4.87(107)***	< 0.001
Depressive symptoms (N = 109)	1.27 (1.35)	0.94 (1.24)	− 1.09(107)	0.28
Anxiety symptoms (N = 109)	1.18 (1.30)	0.97 (1.07)	− 0.81(107)	0.42
Age (N = 109)	37.59 (7.54)	40.79 (7.32)	1.82(107)	0.07
Age of first child (N = 109)	10.09 (5.46)	12.63 (5.41)	1.97(107)	0.05

Frequency displayed as n (%), comparisons via X^2^ or t-Test

## Discussion

In this study, we aimed to investigate parental stress and changes in parental stress during the fourth wave of the COVID-19 pandemic in Germany. Furthermore, addressing the close link between parental stress and physical violence, we aimed to assess the interplay between parental stress and the use of physical violence against children during the pandemic and associated risk factors to identify high-risk families. Our study is, to the best of our knowledge, the first study focusing on parental stress and changes in parental stress as well as physical violence against children and influencing factors during the fourth wave of the pandemic in Germany using a large-scaled population-based sample.

Our results show associations between higher parental stress with the use of physical violence against children, having oneself experienced maltreatment in childhood, mental health symptoms, and certain sociodemographic characteristics. Further, our data shows increased parental stress during the pandemic to be associated with female sex, use of physical violence against children and having experienced more maltreatment in childhood.

In our sample, parents who have ever used physical violence against their children have been characterized by higher parental stress levels, a stronger increase in parental stress during the pandemic, own maltreatment experience during childhood, mental health symptoms and sociodemographic characteristics. Parents who have reported to have used more violence during the pandemic have been characterized by higher parental stress levels, a stronger increase of parental stress during the pandemic, a higher risk of having pre-existing psychiatric disorders, and own maltreatment experience in childhood.

In our sample, the mean parental stress was higher compared to a similar population representative sample during a slightly earlier stage of the pandemic [57]. Although not very comparable as in the study by Kölch and colleagues, the PSS was used with only 17 instead of 18 items, this may point towards a decrease in PSS with relaxations of Corona restrictions, which co-occurs with an improvement in quality of life from earlier to later stages of the pandemic [58]. Concerning changes in parental stress during the pandemic, we observed a slight increase in parental stress on average compared to before the pandemic. This underlines that although there might have been some positive aspects of the pandemic for families, overall, the negative effects outweigh the positive ones, and parents describe themselves to be more stressed during the pandemic compared than before.

Even though about 70% of parents who have ever used physical violence against their children have reported a decrease or no change during the pandemic, about 16% have reported an increase in the use of physical violence. This is in line with previous research, which observed increases between 2 and 20%, depending on the form of physical violence [43]. These results show that there is a small group of children at higher risk for enduring more physical violence during the pandemic. Reconsidering the various negative consequences of physical violence [59–61] this percentage, even if small, is of utmost importance and needs to be characterized better to provide targeted support for these high-risk families.

In our sample, higher levels of parental stress were associated with physical violence against children. Parents reporting to have used more physical violence during the pandemic were characterized by higher parental stress and a stronger increase of parental stress during the pandemic. These findings are in line with recent literature [20, 62, 63], confirming close associations between parental stress and physical violence against children. Stress can affect parental resources, heighten negative coping strategies and increase the risk for parents to vent their negative emotions through e.g. physical violence [64]. Moreover, parents who experience high stress are more likely to have lower parental sensitivity [65, 66], which is linked to child maltreatment [66].

Our data show associations between higher parental stress and depressive symptoms as well as symptoms of anxiety. This link has been shown by others for the time before [67, 68] and during the pandemic [24–26]. Mental health symptoms can go along with emotional instability, making caregiving more difficult. Parents can have difficulties meeting the needs of the child adequately, increasing the risk of neglect and maltreatment [69]. Furthermore, in line with others [45, 46], higher mental health symptoms were associated with the use of physical violence against children during the pandemic. One explanation could be, that with increased mental health symptoms, especially depressive symptoms, more conflicts in the parent–child relationship can arise [70], increasing the risk for physical violence. However, contrary to the literature [46], mental health symptoms were not associated with changes in parental stress and changes in physical violence since the beginning of the pandemic. This is interesting and should be point of further research.

Similarly, pre-existing somatic and psychiatric disorders showed, contrary to others [24], no association to parental stress, and changes in parental stress during the pandemic. One explanation may be that in our analyses, with depressive and anxiety symptoms, the most common disorders are included separately therefore drawing too much variance in the model. However, parents who have ever used physical violence against their children and parents with an increase in the use of physical violence against children had a higher risk of having pre-existing mental disorders. This is in line with others, showing the increased risk for maltreatment in parents with psychiatric disorders [71–73]. Here, moderation and mediation analysis to explore the associations of parental stress, mental health and physical violence could be interesting for future research.

Having experienced more maltreatment during parent’s own childhood was associated with higher levels of parental stress, an increase of parental stress during the pandemic, the use of physical violence against children and an increased use of physical violence during the pandemic. Concerning parental stress, this association has been shown in other research before [74, 75] and during the pandemic [27]. Lotto et al. [74] confirm in their review close associations between on the one hand childhood adversities and parental stress and on the other hand between childhood adversities and later negative parenting. Other research [75] underlines our results regarding a dose–response relationship between the experience of own childhood maltreatment and parental stress. Concerning the use of physical violence against children, our results are in line with previous research [76, 77] showing an association between parental childhood adversities and positive attitudes towards corporal punishment. Taken together, own maltreatment experiences in childhood seem to be an important risk factor for higher parental stress, use of physical violence against children and not being able to cope well with the challenges of the pandemic. Future research could examine these relationships in greater detail, if parental stress could be a mediator to (partly) explain the relationship between own maltreatment experiences in childhood and use of physical violence against children.

In our data, sex was not associated with parental stress levels, suggesting that mothers and fathers may be equally stressed at this stage of the pandemic. This contrasts previous research [23, 24], but may be explained by the different stages of the pandemic. While other studies took place during an early stage of the pandemic, our study took place at a quite late stage of the pandemic, where schools and childcare facilities have reopened [78]. Concerning changes in parental stress during pandemic, in our sample, women reported a stronger increase in parental stress compared to men. This underlines, however, the often-described higher burden for women, especially for mothers, during the pandemic [5, 8, 79–81].

In contrast to others [23, 82] employment and income were not associated with parental stress, changes in parental stress and an increase of physical violence against children during the pandemic in our sample. This is surprising, as economic hardship is a well-known stressor in families, numerously shown to be associated with parental stress levels and physical violence against children [64, 83]. Also, in our data, having ever used physical violence against children was associated with a higher risk of having an income under the poverty level, in line with others [46].

Living in a relationship with the father/mother of the youngest child showed to be a protective factor against higher stress levels, but not for changes in parental stress, use of physical violence or increase in use of violence during the pandemic. While previous studies [22] often investigated the difference between single-parents and parents in a relationship, our study only asked if the parent is living in a relationship with the father/mother of the youngest child, but not if the parent is in a relationship. This may explain the seen differences.

In contrast to other studies [21, 22, 84], but in line with some [83], in our sample, younger age of the child was associated with higher parental stress. In our data, no association regarding age of the first child and pandemic-associated change in parental stress was seen. This could be due to more care and custody duties of parents with younger children. However, in our study, only the age of the first child was considered, existence and age of siblings was not included into analyses. This may limit the validity of our results. Future research could investigate different age groups, e.g., elementary students needing increased support with homeschooling. For example, de Oliveira et al. [85] found two age groups (2–9 and 14–18 years), which are at risk of being victims of violence during the pandemic.

Work-related changes of the parents were not associated with changes in physical violence against children or parental stress. This is in contrast to previous research [22]. Potentially, as our study was conducted during a late stage of the pandemic, working conditions already had normalized again or employees already have adapted to the new situation.

### Strengths and limitations

A strength of this study is that a subsample of a large-scaled, population-based study was used, strengthening the generalizability of our results. Besides, we have used a well-established measure with a wide variance to investigate parental stress and changes in parental stress. On top, we have included many sociodemographic and psychological variables, mental health problems as well as pandemic-related variables like changes in working conditions. However, there are some limitations to consider. In our study, only self-reporting measures were used. These could have been influenced by social desirability, especially when investigating topics like use of physical violence against children. Concerning changes in physical violence this change was estimated by the participants themselves based on one single question. Therefore, potential bias in this subjective estimate might be possible. Future research should investigate this with a more objective measure. On the other hand, these sensitive questions were not asked face-to-face but, in a questionnaire guaranteeing more privacy. Moreover, samples size varied strongly between analyses and for some analyses, sample size was very low. In our study, a cross-sectional design was used, thus, no causality can be concluded. Concerning changes in parental stress, we measure a retrospective recall of these changes. With this operationalization, we cannot capture the fluctuating stress over-time. Future research should therefore capture these changes with longitudinal designs, enabling to compare different stages of the pandemic. Baseline measures are missing. Besides, due to the rather exploratory character of our analyses, we have focused on variables based on our literature research in order to avoid inclusion of too many variables and potential overlapping. However, there are many more possible influencing factors, in particular addressing pandemic-specific information, which weren´t included in our analysis and should be point of further research. Besides these limitations, our study provides important insights in parental stress during the pandemic and characteristics of parents who use physical violence against children during the pandemic in a large population-representative sample in Germany.

## Conclusion

In sum, our data show close associations between parental stress during the pandemic with physical violence against children during the pandemic. These results underline the relevance of parental stress for the prevention of child maltreatment, more so during times of crises such as the pandemic. Interventions aiming to reduce physical violence against children should obligatorily include measures to reduce parental stress. Besides sociodemographic factors like age of the parent and relationship status, parent’s own history of child maltreatment experience and mental health symptoms are significantly linked to parental stress. Thus, targeted support for parents with mental health problems should be included in prevention programs of parental stress and physical violence against children. Parental maltreatment experiences in childhood showed significant associations to increased parental stress during the pandemic. Therefore, members of the help-systems for children should include asking for parental maltreatment experiences, to identify this risk group and provide adequate support to break the cycle of violence. However, our analyses have a rather exploratory character and besides the strength of our large scale representative sample. Thus, further research focusing further assessing the interplay between parental stress and physical violence against children during the pandemic, is needed. Future research should use prospective designs and focus on the development of intervention and prevention programs for parental stress to further prevent child maltreatment.

## Supplementary Information

**Additional file 1**: **Table S1.** Multicollinearity statistics for the regression model with PSS as dependent variable. **Figure S1.** Distribution of residuals from the regression model with PSS as dependent variable (M=0, SD=1, N=415). **Table S2.** Multicollinearity statistics for the regression model with change in parental stress as dependent variable. **Figure S2.** Scatterplot of residuals for the regression model with PSS as dependent variable. **Figure S3.** Distribution of residuals from the regression model with change in PSS as dependent variable (M=0, SD=1, N=293). **Figure S4.** Scatterplot of residuals for the regression model with change in parental stress as dependent variable.

## Data Availability

The data presented in this study are available on reasonable request from the corresponding author.

## Abbreviations

ACEs: Adverse childhood experiences
ADM: Arbeitskreis markt- und sozialforschungsinstitute e. V.
PSS: Parental stress scale
PHQ2: Patient health questionnaire 2
GAD2: Generalized anxiety disorder 2
HC3: Heteroscedasticity-consistent 3
M: Mean
SD: Standard deviation
B: Beta coefficient
SE: Standard error
