# Supplementary material for: Parental stress and physical violence against children during the second year of the COVID-19 pandemic: results of a population-based survey in Germany
Source: Child Adolesc Psychiatry Ment Health. 2023 Feb 20;17:25. doi: 10.1186/s13034-023-00571-5 (PMC9940081; doi:10.1186/s13034-023-00571-5)

**Additional file 1**

**Table S1**

*Multicollinearity statistics for the regression model with PSS as dependent variable.*

| Variable | Variance Inflation Factor (VIF) |
| --- | --- |
| Femal gender | 1.07 |
| Employment | 1.11 |
| Income under poverty level | 1.15 |
| Living in relationship with father/mother of youngest child | 1.10 |
| Age of first child | 1.02 |
| Pre-existing somatic disorder | 1.11 |
| Pre-existing psychiatric disorder | 1.21 |
| Depressive symptoms | 1.76 |
| Anxiety symptoms | 1.87 |
| Use of physical violence against children | 1.04 |
| Parental experience of child maltreatment | 1.15 |

*Variance Inflation factors above 10 represent strong evidence for multicollinearity. Durbin Watson statistic=1.66(values below 0.7 and above 2.3 indicate autocorrelation of residuals).*

**Figure S1**

*Distribution of residuals from the regression model with PSS as dependent variable (M=0, SD=1, N=415)*


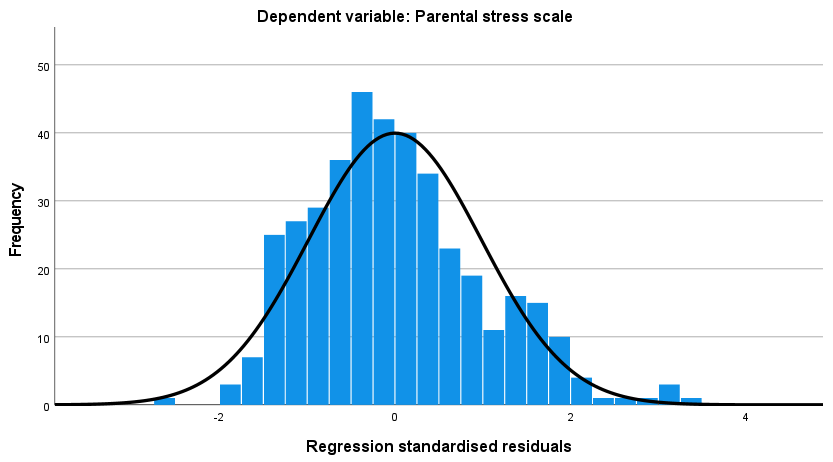


**Figure S2**

*Scatterplot of residuals for the regression model with PSS as dependent variable*


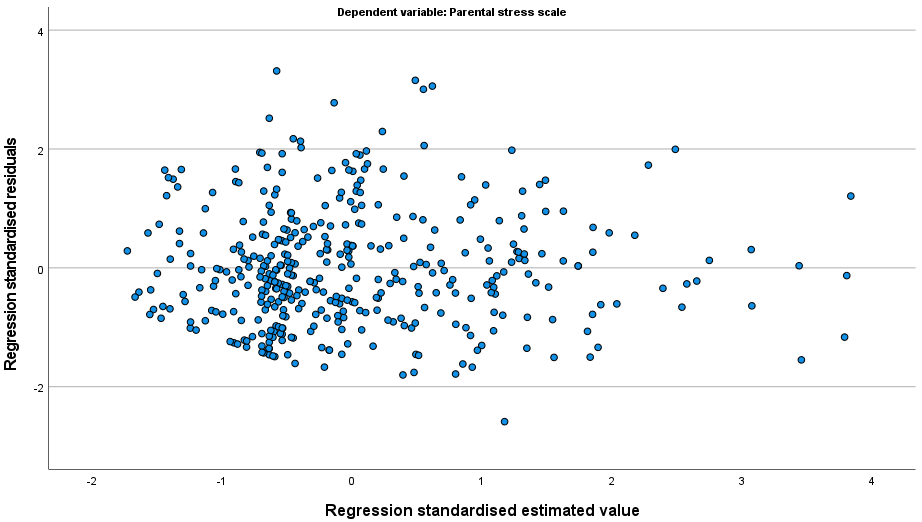


**Table S2**

*Multicollinearity statistics for the regression model with change in parental stress as dependent variable.*

| Variable | Variance Inflation Factor (VIF) |
| --- | --- |
| Femal gender | 1.15 |
| Employment | 1.10 |
| Income under poverty level | 1.09 |
| Living in relationship with father/mother of youngest child | 1.11 |
| Age of first child | 1.05 |
| Pre-existing somatic disorder | 1.07 |
| Pre-existing psychiatric disorder | 1.27 |
| Depressive symptoms | 1.72 |
| Anxiety symptoms | 1.81 |
| Use of physical violence against children | 1.05 |
| Parental experience of child maltreatment | 1.27 |
| „System-relevant“ job | 1.31 |
| Working from home | 1.27 |
| „System-relevant“ job partner | 1.20 |
| Working from home | 1.20 |

*Variance Inflation factors above 10 represent strong evidence for multicollinearity. Durbin Watson statistic=1.53(values below 0.7 and above 2.3 indicate autocorrelation of residuals).*

**Figure S3**

*Distribution of residuals from the regression model with change in PSS as dependent variable (M=0, SD=1, N=293)*


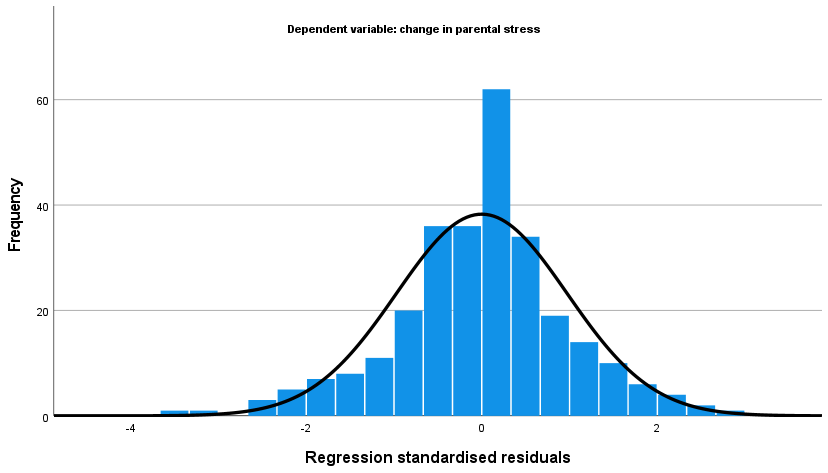


**Figure S4**

*Scatterplot of residuals for the regression model with change in parental stress as dependent variable*


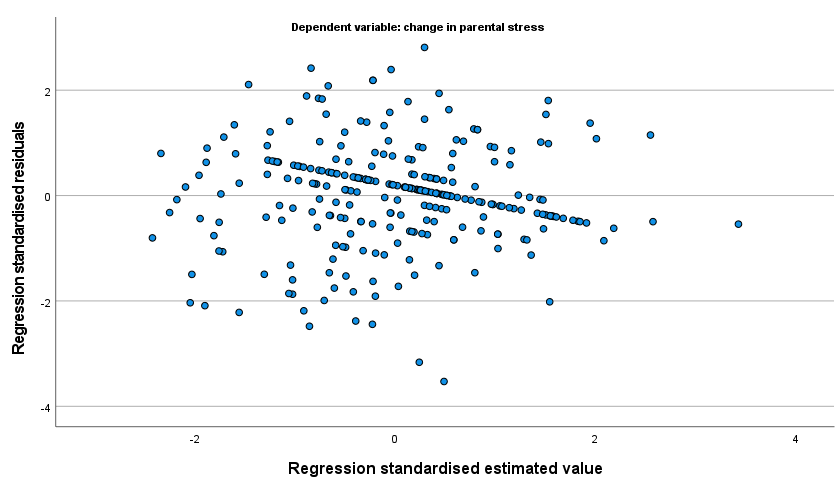

Supplement: Supplementary file 1 — Additional file 1: Table S1. Multicollinearity statistics for the regression model with PSS as dependent variable. Figure S1. Distribution of residuals from the regression model with PSS as dependent variable (M=0, SD=1, N=415). Table S2. Multicollinearity statistics for the regression model with change in parental stress as dependent variable. Figure S2. Scatterplot of residuals for the regression model with PSS as dependent variable. Figure S3. Distribution of residuals from the regression model with change in PSS as dependent variable (M=0, SD=1, N=293). Figure S4. Scatterplot of residuals for the regression model with change in parental stress as dependent variable. [file 13034_2023_571_MOESM1_ESM.docx]
